# Stress perfusion cardiac MRI with regadenoson and gadofoveset trisodium

**DOI:** 10.1186/1532-429X-17-S1-P113

**Published:** 2015-02-03

**Authors:** Bradley D Allen, Neil Chatterjee, Jad Bou Ayache, Benjamin H Freed, Daniel C Lee, Timothy Carroll, Michael Markl, Jeremy D Collins, James C Carr

**Affiliations:** Radiology, Northwestern University, Chicago, IL USA; Medicine-Cardiology, Northwestern University, Chicago, IL USA; Biomedical Engineering, Northwestern University, Chicago, IL USA

## Background

Stress perfusion cardiac MRI (CMR) with a high relaxivity blood-pool contrast agent should allow for improved quantification of myocardial perfusion and myocardium blood volume in patients with coronary artery disease (CAD). In the current study, we sought to evaluate the diagnostic performance and image quality of stress perfusion CMR using the intravascular contrast agent gadofoveset trisodium and regadenoson stress agent in patients with suspected CAD.

## Methods

Patients with suspected CAD were identified based on referral for cardiac catheterization and/or stress testing and recruited to undergo CMR at 1.5T (MAGNETOM Avanto, Siemens, Erlangen, Germany). The CMR protocol included stress perfusion imaging using 5 ml intravenous (IV) regadenoson (Lexiscan, Astellas US LLC) as the stress agent, and the effects of regadenoson were reversed with 50 mg IV aminophylline following the completion of stress imaging. Left ventricular (LV) base, mid, and apex short axis cine images were acquired in identical slice positions during first pass using 5 ml IV gadofoveset trisodium contrast (Ablavar, Lantheus) at both stress and rest. Perfusion images were reviewed and sensitivity and specificity for CAD diagnosis were calculated. Overall image quality, image noise, and presence of artifact for each patient scan were graded on a scale of 1 - poor to 5 - excellent.In addition to the perfusion scan, the MRI protocol included pre- and post-contrast 2D Modified Look-Locker (MOLLI) measurement of T1 which were used to calculate the myocardial blood volume (in ml/100g of tissue).

## Results

**Figure 1:**
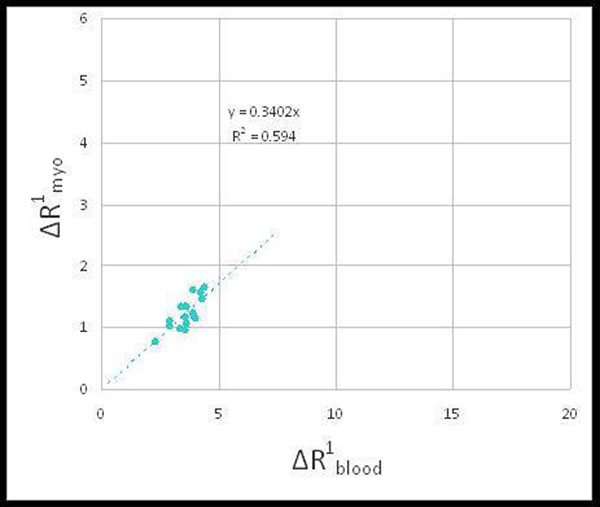
Figure 1

Stress perfusion CMR was performed on n = 20 subjects (age: 61.8 ± 12.2 years, M:F = 13:7). Seventeen patients (85%) had cardiac catheterization within 1 month of CMR, while 2 patients had a positive nuclear stress test, and 1 patient had positive stress echocardiography. Seven catheterized patients (41%) had ≥ 70% occlusion of at least one of the left anterior descending, left circumflex, or right main coronary artery. The sensitivity/specificity for diagnosing CAD on a per-patient basis was 0.8/0.5. Qualitative assessment revealed good image quality (4.38 ± 0.46), low noise (4.45 ± 0.28), and little artifact (4.28 ± 0.38).For pre-/post- stress perfusion MRI in our cohort, the average ΔR_1_^myo^ was 1.23 ± 0.24 msec^-1^ while the average ΔR_1_^blood^ was 3.63 ± 0.54 msec^-1^. As shown in figure 1, the slope of the linear regression between ΔR_1_^myo^ and ΔR_1_^blood^ was a = 0.34 suggesting that gadofoveset trisodium does not behave as a true intravascular agent in the myocardium.

## Conclusions

Stress perfusion CMR using regadenoson and gadofoveset trisodium provides diagnostic quality images with sensitivity for CAD diagnosis comparable to published results. Our results suggest that gadofoveset trisodium does not behave as a true intravascular agent in the myocardium and thus a quantification of myocardial intravascular blood volume (and hence a calculated coronary flow reserve) cannot be calculated using the approach.

## Funding

Research support for this study was provided by Astellas US LLC.

